# A Systematic Review of Clinical Functional Outcomes After Medial Stabilized Versus Non-Medial Stabilized Total Knee Joint Replacement

**DOI:** 10.3389/fsurg.2018.00025

**Published:** 2018-04-11

**Authors:** Tony Young, Michelle M. Dowsey, Marcus Pandy, Peter F. Choong

**Affiliations:** ^1^Department of Surgery, University of Melbourne, St. Vincent’s Hospital Melbourne, Fitzroy, VIC, Australia; ^2^Department of Orthopaedic Surgery, St. Vincent’s Hospital Melbourne, Fitzroy, VIC, Australia; ^3^Department of Mechanical Engineering, Melbourne School of Engineering, University of Melbourne, Parkville, VIC, Australia

**Keywords:** outcome, clinical function, knee prosthetic design, osteoarthritis, patient reported outcome measure

## Abstract

**Background:**

Medial stabilized total knee joint replacement (TKJR) construct is designed to closely replicate the kinematics of the knee. Little is known regarding comparison of clinical functional outcomes of patients utilising validated patient reported outcome measures (PROM) after medial stabilized TKJR and other construct designs.

**Purpose:**

To perform a systematic review of the available literature related to the assessment of clinical functional outcomes following a TKJR employing a medial stabilized construct design.

**Methods:**

The review was performed with a Preferred Reporting Items for Systematic Review and Meta-Analyses (PRISMA) algorithm. The literature search was performed using variouscombinations of keywords. The statistical analysis was completed using Review Manager (RevMan), Version 5.3.

**Results:**

In the nineteen unique studies identified, there were 2,448 medial stabilized TKJRs implanted in 2,195 participants, there were 1,777 TKJRs with non-medial stabilized design constructs implanted in 1,734 subjects. The final mean Knee Society Score (KSS) value in the medial stabilized group was 89.92 compared to 90.76 in the non-medial stabilized group, with the final KSS mean value difference between the two groups was statistically significant and favored the non-medial stabilized group (SMD 0.21; 95% CI: 0.01 to 0.41; *p* = 004). The mean difference in the final WOMAC values between the two groups was also statistically significant and favored the medial stabilized group (SMD: −0.27; 95% CI: −0.47 to −0.07; *p* = 0.009). Moderate to high values (*I^2^*) of heterogeneity were observed during the statistical comparison of these functional outcomes.

**Conclusion:**

Based on the small number of studies with appropriate statistical analysis, we are unable to reach a clear conclusion in the clinical performance of medial stabilized knee replacement construct.

**Level of Evidence:**

Level II

## Introduction

### Rationale

The most effective remedy for end stage osteoarthritis is a total knee joint replacement (TKJR). Demand for this procedure is expected to grow as high as 3.48 million procedures per year by 2030 in the United States alone (1). It is generally associated with excellent longevity and survivorship −92% at 16 years (2). It provides reliable pain relief and restoration of moderate function of daily activities for patients suffering from severe joint degeneration. In Australia, the use of primary TKJR continues to increase with 50,623 TKJR procedures performed in 2015 (3), and an additional 52,836 TKJR proceduresin 2016 (2). In 2016, there were 2.8% more TKJR procedures than 2015 and 139.8% more than in 2003 (2). As a proportion of all knee replacement procedures, primary TKJR increased from 76.7% in 2003 to 87.0% in 2016 (2).Osteoarthritis is the most common diagnosis for primary total knee replacement (97.6%) (2).

Due to the ongoing pursuit of optimising the longevity and performance of the prosthesis, there are many prosthetic designs available (4). These constructs have emerged based on many published *in vivo* studies of the knee motion, as well as biomechanical theories of knee kinematics such as single radius (5), multi radii (6), fixed–bearing (7), mobile–bearing (8), posterior stabilized (9), cruciate retaining (10), and cruciate sacrificing (11). In Australia alone, there have been 119 femoral and tibial prosthesis combinations used in primary TKJR reported to the National Joint Replacement Registry (2, 3).

The paradigm of enhanced medial stabilizer was based on the physiological (4, 12), as well as *in vivo* knee kinematic observations reported in studies (13, 14). These studies reported that the knee joint flexes with minimal anteroposterior motion in the medial tibio-femoralcompartment, while the lateral femoral condyle travels anteroposteriorly rotating about the center of the medial compartment thus producing a “medial pivot” motion (12–14).

**Figure 1:**
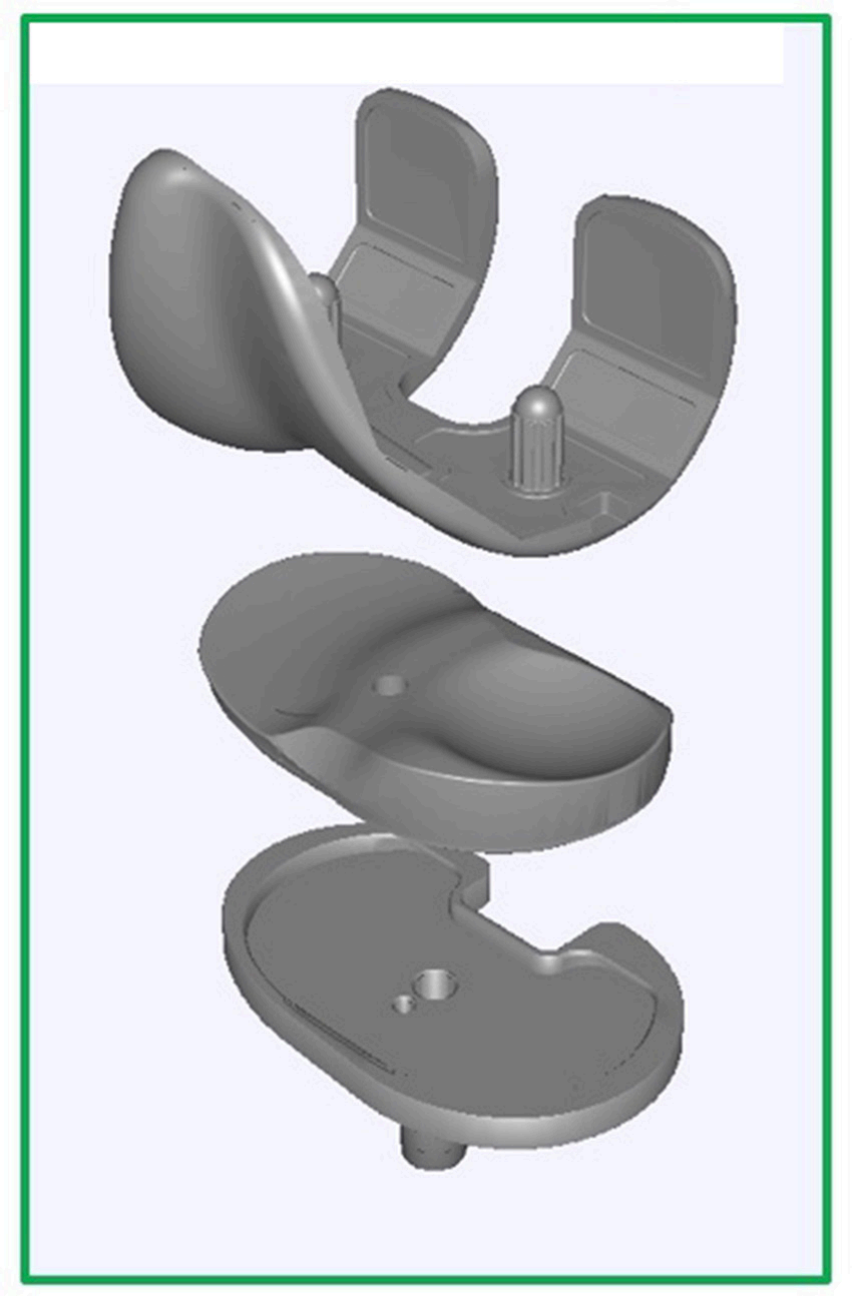
Medial Stabilized Total Knee Joint Replacement Construct Design (16).

The medial stabilized femoral component employs a single radius curvature design to the distal and posterior femur (15). The tibial insert is asymmetric with a highly conforming medial compartment and a “dish like” lateral compartment allowing unrestricted anteroposterior motion as shown in Figure 1. This is known as the medially conforming “ball-and-socket” construct (15). The conforming medial articular spherical surface permitsinternal rotation of the tibia on femur around a medial axis as the knee construct flexes (4, 15), and allows posterior rolling and sliding of the lateral femoral condyle around a stable spinning medial femoral condyle during knee flexion. The epicondylar axis of the femur serves as the axis of rotation of the medial stabilized implant. In theory, these design features would lower the contact stresses on the tibial surface, providing for enhanced durability of the polyethylene (15), and improved forces within the quadriceps especially in early flexion (17). Furthermore, some studies have shown that the medial stabilized design provides good anteroposterior stability throughout the range of motion whilst the spherical shape permits tibial-femoral rotation around a medial axis, minimising condylar lift-off during knee flexion (4, 15, 18).

### Objectives

The overall goal of this study was to perform a systematic review of the available literature related to the assessment of clinical functional outcomes following a total knee joint replacement (TKJR) employing a medial stabilized construct design with a focus on Patient Reported Outcome Measures (PROM).

### Research Question

Our specific aim was to determine whether differences exist in PROMs between patients with a medial stabilized TKJR construct and those with non-medial stabilized designs.

## Methods

### Study Design, Systematic Review Protocol and Search Strategy

**Figure 2:**
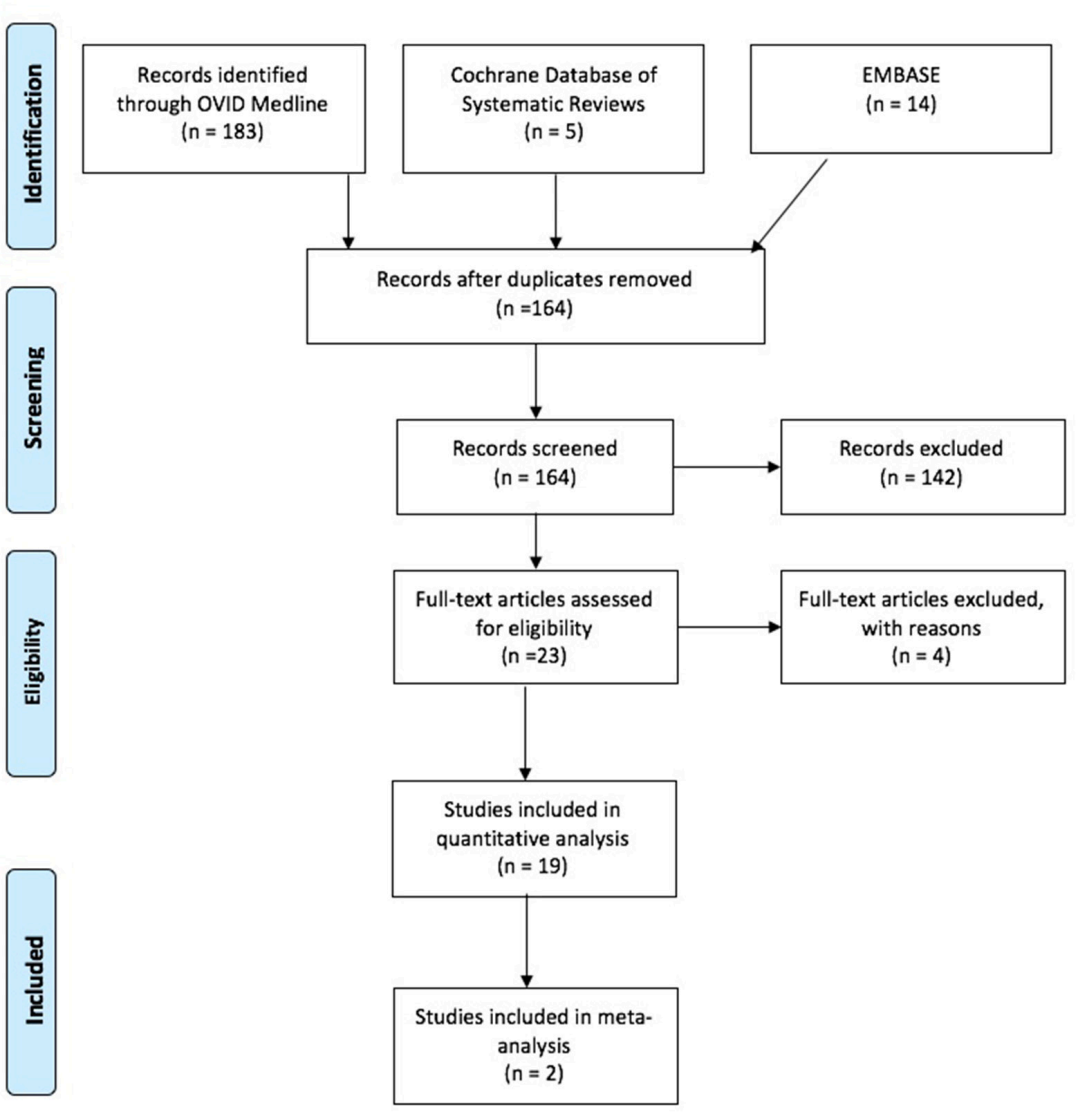
PRISMA (2009) Flow Diagram.

A systematic review of the literature was performed with a Preferred Reporting Items for Systematic Reviews and Meta-Analyses (PRISMA) (19) checklist and algorithm. The search algorithm in accordance with PRISMA is shown in Figure 2. An electronic search was performed with OVID Medline, Embase and Cochrane database of systematic reviews since the inception of these respective databases up until 5 July 2016. The search strategy is shown in Figure 2. Reference sections of all identified papers were examined for any undetected studies.

1. Studies were included when they fulfilled the following inclusion criteria:
  - Studies reporting TKJR surgery with the use of a congruent medial tibiofemoral articulationaimed at replicating a medial stabilized kinematic pattern;
  - Studies reporting clinical outcome measures including patient’s subjective measures, and objective function tests such as knee range of motion.

Case reports, literature reviews, conference notes, letters to editor, posters, or any non-English language articles and studies involving revision procedures were excluded. Furthermore, studies that only examined radiographic outcomes, *in vitro* kinematics, intraoperative kinematics or *in vivo*kinematics were also discarded. Twenty studies met the inclusion criteria, one further study was excluded as it examined the relative clinical performance of two variations of a medial stabilized TKJR construct. Each included study was carefully scrutinisedfor its methodology, and the following data were extracted: study design; type of knee implant used; follow-up periods; types of validated clinical function assessments (for example KSS, KSFS, OKS, ROM and radiographic parameters); and anthropometrics (age, BMI).

### Data Extraction

**Figure 3:**
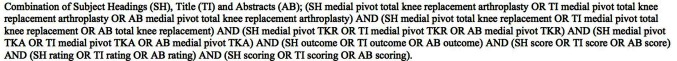
Search Strategy for Published Peer Reviewed Articles (OVID Medline, Cochrane Database of Systematic Reviews, and PubMed).

The literature search resulted in a total of 202 articles with the parameters documented in Figure 3. 19 duplicates had been identified and removed, leaving 164 abstracts to be scrutinized. 164 articles were screened, of which 142 were deemed unsuitable due to off-topic abstracts, failure to satisfy the inclusion criteria, or both (Figure 3). After studying the remaining 23 articles, 19 studies were identified from the references of the full-text articles and manually included in the analysis (Table 1). Of the seven comparison studies, some critical statistical details such as SD was absent and hence only two formed part of the quantitative analysis.

**Table 1:** Identified Studies.

**Author(s)**	**Journal**	**Study Design and Level of Evidence**	**Methods**	**Subjects Demographics**					**Primary Outcome Measure**	**Other Outcome Measures**	**Follow Up Period; months (SD)**	**Follow up Period Range, months**	**Prosthesis Used in Study**
				**Number of Patients/Number of Knees in Study**	**Mean Age, years (SD)**	**Range, years**	**Gender M: F**	**BMI (kg.m^2^)**					
Anderson et al. (20)	*J Surg Orthop Adv*	CohortLevel II	*Investigate the clinical, radiographic outcome measures in subjects with medial pivot construct TKJR*	189 patients (204 knees)	69 (−)	39–87	111M: 165F	Not reported	ROM, KSS	Survivorship, radiology	64.8 (−)	60–91.2	ADVANCE®
Bae et al. (21)	*Arthroplasty*	Prospective cohort comparisonLevel II	*Comparison of clinical and radiographic results between patients with medial pivot and posterior stabilised prosthesis*	**MP Group** = 125 patients (150 knees)	66.7 (7.1)	42–83	4M: 121F	26.4 (3.2)	KSS, WOMAC, Kujala score, Feller scoring system, ROM	Radiology	62.4 (32.4)	24–152.4	ADVANCE®
				**PS Group** = 138 patients (150 knees)	66.7 (6.5)	Not reported	2M: 136F	25.9 (4.4)			61.2 (43.2)	Not reported	PFC® (PS)
Brinkman et al. (22)	*ANZ J Surg*	Prospective cohortLevel II	*Compare pre and postoperative subjective clinical outcome measure data, radiographic measurements, and survivorship cross referenced against AOA NJRR data*	47 patients (50 knees)	69 (−)	45–82	35M: 12F	Not reported	KSS, WOMAC, subjective functional score, ROM	Radiology, survivorship, complications	119.52 (−)	20.52–168	ADVANCE®
Chinzei et al. (23)	*The Knee*	Retrospective cohortLevel II	*Investigate subjective clinical outcome measure and radiographic measurements in those with medial pivot TKJR 8 years after implantation*	76 patients (85 knees)	70.2 (8.1)	51–88	5M: 71F	26.5 (4.6)	KSS	Radiology	93.1 (14.3)	72–132	ADVANCE®
Cho et al. (24)	*Orthopaedics*	Prospective cohortLevel II	*Report clinical outcome through subjective functional sores and radiographic posterior femoral condylar translation through progressive knee flexion*	30 patients (30 knees)	Not reported	Not reported	Not reported	Not reported	KSS, ROM	Radiology	24 (−)	Not reported	ADVANCE®
Fan et al. (25)	*Arthroplasty*	Prospective cohortLevel II	*Investigate clinical and radiographic outcomes of subjects with medial pivot TKJR implanted 5 years after implantation*	55 patients (58 knees)	65.1 (−)	48–83	13M: 42F	Not reported	ROM, KSS	Survivorship, radiology	64.7 (−)	Not reported	ADVANCE®
Hossain et al. (26)	*Clin Orthop Relat Res*	Randomised Control Trial Level I	*Comparison of subjective functional scores and clinical ROM in patients with medial pivot TKJR construct to those with a posterior stabilised construct design*	**MP Group** = 40 patients (40 knees)	72.5 (9.7)	53–88	9M: 31F	28.9 (6.2)	ROM	KSS, WOMAC, OKS, SF-36, TKFQ	24 (−)	Not reported	
				**PS Group** = 40 patients (40 knees)	68.9 (12.1)	44–84	18M: 22F	29.5 (8.1)					PFC® (PS)
Iida et al. (27)	*Knee Surg Sports Traumatol Arthrosc*	CohortLevel II	*Subjective clinical measures, ROM, radiographic measurements, and survivorship data in patients with alumina medial pivot TKJR*	80 patients (107 knees)		45–86	4M: 76F	Not reported	ROM, KSS	Radiology, survivorship	60 (−)	12–84	MPK Alumina Femur
Ishida et al. (28)	*Knee Surg Sports Traumatol Arthrosc*	Randomised Control TrialLevel I	*Investigate and compare the clinical and radiographic measures between patients with double-high tibial insert and medial pivot tibial insert*	**MP Group** = 20 patients (20 knees)	71 (−)	60–81	5M: 15F	26.0 (−)	KSS, ROM, KSFS, UCLA	Nil	57 (−)	48–62	ADVANCE®
				**DH Group** = 20 patients (20 knees)	72 (−)	63–79	5M: 15F	27.2 (−)			57 (−)	48–61	
Karachalios et al. (29)	*The Knee*	Prospective cohortLevel II	*Clinical outcome with subjective clinical measures and radiographic measurements in patients with TKJR with medial pivot construct design*	225 patients (284 knees)	71 (−)	52–84	41M: 184F	Not reported	ROM, KSS, WOMAC, SF - 12, OKS	Radiology, survivorship	80.4 (−)	48–108	ADVANCE®
Kim et al. (30)	*Clin Orthop Relat Res*	Prospective cohort comparisonLevel II	*Investigate and compare the clinical and radiographic measure outcomes in patients with bilateral TIKJR implants: fixed bearing (medial pivot) in one knee, and mobile bearing (medical pivot) in one knee, and mobile bearing (PFC) in the other*	**MP Group** = 92 patients (92 knees)	69.5 (7.92)	55–81	7M: 85F	27.8 (3.15)	ROM, KSS, HSSKS	Radiology, survivorship	31.2 (−)	24–36)	ADVANCE®
				**PFC Group** = 92 patients (92 knees)									PFC®
Moonot et al. (31)	*Knee Surg Sports Traumatol Arthrosc*	Retro - pro cohortLevel II	In vivo kinematic analysis of kneeling and lunging activities after TKJR performed	13 patients (15 knees)	75 (7)	61–86	4M: 9F	32 (5)	OKS, KSS, IKS	Radiology, fluoroscopy	17 (4)	13–27	MRK™
Pritchett (32)	*JBJS (Br)*	Prospective cohortLevel II	*Outcome assessed using subjective clinical measures in patients with TKJRs*	**ACL - PCL Group** = 201 patients (201 knees)	66 (−)	45–89	103M: 241F	Not reported	KSS, ROM, “which knee feels better”	Radiology	99.6 (−)	24–168	ADVANCE®
				**PCL Group** = 199 patients (199 knees)	71 (−)						110.4 (−)		
				**MP Group** = 142 patients (142 knees)	67 (−)						48.0 (−)		
				**PS Group** = 146 patients (146 knees)	70 (−)						79.2 (−)		
Pritchett (17)		Prospective cohortLevel II	*Follow-up study examining clinical and radiographic outcomes of patients with bilateral TKJRs with different construct designs*	**ACL - PCL Group** = 201 patients (201 knees)	68 (−)	45–89	132M: 308F	Not reported	Not reported	“Which knee feels better”	99.6 (−)	24–168	ADVANCE®
				**PCL Group** = 205 patients (205 knees)							110.4 (−)		
				**PS Group** = 152 patients (152 knees)							79.2 (−)		
				**MB Group** = 83 patients (83 knees)							43.2 (−)		
				**MP Group** = 239 patients (239 knees)							73.2 (−)		
Schmidt et al. (33)	*International Orthopaedics*	Prospective cohortLevel II	Clinical outcomes and radiographic measurements in patients with medial pivot TKJR 5 years after implantation	320 patients (365 knees)	66.5 (−)	29–86	258M: 107F	Not reported	KSS, ROM	Radiology, survivorship, complications	63.6 (−)	24–130.8	ADVANCE®
Shakespeare et al. (34)	*The Knee*	Retro - pro cohort comparison studyLevel II		**MP Group** = 248 patients (261 knees)	76 (−)	Not reported	51%: 49%	Not reported	ROM	Nil	12 (−)		ADVANCE®
				**PS Group** = 257 patients (288 knees)	78 (−)	Not reported	48%: 52%	Not reported					The 413 PS Prosthesis
Shimmin et al. (35)	*The Knee*	Prospective cohortLevel II	*Analysis of knee kinematics by video fluoroscopy during four different weight-bearing activities (pivoting/lunging/step-up/step-down).*	14 patients (14 knees)	69 (−)	51–83	7M: 7F	Not reported	Fluoroscopic kinematics	OKS, KOOS, Kujala score, EuroQol	34 (−)	30–36	SAIPH®
Vecchini et al. (36)	*The Knee*	Prospective cohortLevel II		160 patients (172 knees)	71 (−)	31–85	42M: 118F	Not reported	KSS, ROM	Radiology, survivorship	84 (−)	48–120	ADVANCE®
Youm et al. (37)	*Knee Surg Sports Traumatol Arthrosc*	Retrospective cohortLevel II		80 patients (120 knees)	66.4 (−)	42–83	9M: 71F	Not reported	ROM, KSS, WOMAC	Survivorship, radiology, complications	64.7 (−)	60–86	ADVANCE®

SD, Standard Deviation; M, Male; F, Female; BMI, Body Mass Index; kg.m^2^, kilogram per meter squared; DH, Double High; MB, Mobile Bearing; MP, Medial Pivot; PS, Posterior Stabilized; KOOS, Knee Injury and Osteoarthritis Outcome Score; VR-12, Veterans Rand - 12.

In the 19 studies identified, there were two randomized controlled trials (26, 28), two retrospective studies (23, 37), and fifteen prospective cohort studies (17, 20–22, 24–27, 29–36). Almost all studies utilised a spectrum of validated functional scores, such as the Knee Society Score (KSS), Western Ontario and McMaster Universities Arthritis Index Score (WOMAC), and Oxford Knee Score (OKS) as primary outcome measures to quantify clinical function (17, 20–33, 35–37). All studies employed clinical examination findings of range of motion (ROM) of the knee as an outcome measure. Shakespeare et al. was the only study that used ROM as the outcome measure alone (34). 13 studies reported on their radiological outcomes in their respective study populations – including alignment of the limb, positioning of the prosthesis components; as well as the presence of signs of radiographic loosening (20–25, 27, 29–33, 36, 37). Nine studies reported on complications and survivorship as part of the outcome measures (20, 22, 25, 27, 29, 30, 33, 36, 37). In addition to using the Knee Society Score (KSS), Pritchett asked the respective study cohorts who have undergone bilateral TKJRs with different prostheses “which knee is better?” (17, 32).

### Data Analysis

Data were analysed using Review Manager (RevMan), Version 5.3. Copenhagen: The Nordic Cochrane Centre, The Cochrane Collaboration, 2014. Mean and standard deviations were extracted from each study to generate forest plots for the meta-analysis. In all studies, *p* < 0.05 was considered statistically significant. The total fixed-effects model was used when quoting all Confidence Intervals (CI) and *p* values.

Calculations were performed comparing participants implanted with a medial stabilized TKJR construct to those implanted with another type of TKJR design in terms of clinical function such as Knee Society Score (KSS), Knee Society Function Score (KSFS), Oxford Knee Score (OKS), Western Ontario and McMaster Universities Arthritis Index (WOMAC), knee range of motion (ROM) and radiographic parameters. An *I^2 ^*was calculated to measure heterogeneity, which represents the percentage of variation in our meta-analysis that is caused by heterogeneity rather than by chance. A low *I^2 ^*value would be 25% or lower and a high *I^2 ^*value to be 75% or higher.

## Results

Table 1: Demographics.

The review included 2448 medial stabilized TKJRs implanted in 2195 participants. The mean age for the medial stabilized group was 69.48 years with the range of 29 (33) to 89 years (17). There were 1,777 TKJRs that were non-medial stabilized designs implanted in 1,734 subjects. The mean age for this group was 75.56 years ranging from 42 (21) to 84 years (26). The mean BMI of the medial stabilized group was 27.18 kg.m^2^ (range 17.8 to 38.9 kg.m^2^), compared to 27.09 kg.m^2^ for the non-medial stabilizedgroup (range 21–41.6 kg.m^2^).

## Clinical Function Parameters

All studies in this literature review reported knee ROM as part of their clinical outcome measures (Table 2); 18 studies (89.5%) employed combination of KSS and KSFS to measure clinical outcome (17, 20–33, 36, 37) (Table 3); 14 (73.7%) reported on radiographic findings in their respective studies (17, 21, 23–28, 30–32, 36, 37) (Table 4). WOMAC was reported in five studies (26.3%) (21, 22, 26, 29, 37), and four used OKS (21.0%) (26, 29, 31, 35) (Table 5). Two studies (10.5%) employed Kujala Knee Scale (21, 35), and Short Form 36 questionnaire (SF-36) (26, 29) as means to quantify clinical outcomes (Table 6). One study used Total Knee Function Knee Questionnaire (TKFQ) (27) (Table 6), one study used a combination of OKS, Knee Injury and Osteoarthritis Outcome Score (KOOS), Kujala knee score, Veterans RAND 12 item health survey (VR-12), and European Quality of Life Scale (EuroQol) to quantify clinical outcomes (33) (Table 7). The Feller Knee Score was used in one study (21) (Table 7), and University of California Los Angeles Activity Score (UCLA) was used in one study (21) (Table 8). There were eight comparison studies (18, 19, 23, 25, 27–30) (42.1%), however only two could be used for the meta-analysis (21, 26). This was due to the lack of critical statistical detail such as the SD to accompany each mean value, which disqualified most studies from inclusion in the meta-analysis.

**Table 2:** Summary of Knee Clinical Range of Motion in Studies.

**Author(s)**		**Knee Range of Motion; ° (SD)**											
		**Flexion Contracture Preop**	**Range**	**Flexion Contracture Postop**	**Range**	**Flexion Contracture Improvement**	**Range**	**ROM Preop**	**Range**	**ROM Postop/Final**	**Range**	**Improvement**	**Range**
Anderson et al. (20)		−	−	−	−	−	−	107 (−)	−	121 (−)	−	14 (−)	−
Bae et al. (21)	**MP Group**	6.2 (6.1)	−	0.8 (2.1)	−	−	−	115.1 (16.7)	−	123.7 (14.8)	−	8.6 (16.7)	−
	**PS Group**	8.2 (11.1)	−	1.0 (3.3)	−	−	−	118.5 (26.7)	−	127.1 (16.1)	−	8.6 (20.7)	−
Brinkman et al. (22)		−	−	−	−	−	−	−	−	110.0 (−)	−	−	−
Chinzei et al. (23)		−	−	−	−	−	−	94.2 (−)	20–140	110.6 (−)	60–130	−	−
Cho et al. (24)		−	−	−	−	−	−	105.5 (11.2)	−	109.3 (9.8)	−	−	−
Fan et al. (25)		−	−	−	−	−	−	103.5 (2.0)	−	115.4 (1.8)	−	−	−
Hossain et al. (26)	**MRK Group**	−	−	−	−	−	−	97.3 (15.3)	50–120	114.9 (12.8)	90–140	−	−
	**PFC Group**	−	−	−	−	−	−	93.9 (19.0)	20–115	100.1 (15.9)	45–110	−	−
Iida et al. (27)								104 (23)	−	114 (20)	−	−	−
Ishida et al. (28)	**MP Group**	−	−	−	−	−	−	110 (−)	85–130	110 (−)	90–130	Median = 0 (−)	−
	**DH Group**	−	−	−	−	−	−	110 (−)	75–135	115 (−)	95–130	Median = 5 (−)	−
Karachalios et al. (29)		−	−	−	−	−	−	101 (−)	70–125	117 (−)	85–135	−	−
Kim et al. (30)	**MP Group**	−	−	−	−	−	−	124 (−)	60–150	115 (−)	80–145	−9 (−)	−
	**PFC Group**	−	−	−	−	−	−	124 (−)	50–150	127 (−)	85–145	3 (−)	−
Moonot et al. (31)		−	−	−	−	−	−	−	−	106 (-)	100–120	−	−
Pritchett (32)	**ACL Group**	−	−	−	−	−	−	−	−	119 (−)	−	−	−
	**MP Group**	−	−	−	−	−	−	−	−	121 (−)	−	−	−
	**PCL Group**	−	−	−	−	−	−	−	−	119 (−)	−	−	−
	**PS Group**	−	−	−	−	−	−	−	−	111 (−)	−	−	−
Pritchett (17)	**ACL − PCL Group**	−	−	−	−	−	−	−	−	119 (−)	−	−	−
	**PCL Group**	−	−	−	−	−	−	−	−	121 (−)	−	−	−
	**PS Group**	−	−	−	−	−	−	−	−	120 (−)	−	−	−
	**MB Group**	−	−	−	−	−	−	−	−	124 (−)	−	−	−
	**MP Group**	−	−	−	−	−	−	−	−	125 (−)	−	−	−
Schmidt et al. (33)		−	−	−	−	−	−	115 (−)	−	119 (−)	−	4 (−)	−
Shakespeare et al. (34)	**MP Group**	−	−	−	−	−	−	112 (−)	−	111 (−)	−	−1 (−)	−
	**PS Group**	−	−	−	−	−	−	109 (−)	−	109 (−)	−	0 (−)	−
Shimmin et al. (35)		−	−	−	−	−	−	−	−	127 (13)	100–155	−	−
Vecchini et al. (36)		−	−	−	−	−	−	97.7 (1.36)	60–130	112.5 (1.76)	75–130	−	−
Youm et al. (37)		7.6 (−)	−	1.5 (−)	−	−	−	107.5 (−)	−	119.0 (−)	−	5.4 (−)	−

DH, Double High; MB, Mobile Bearing; MP, Medial Pivot; PS, Posterior Stabilized.

**Table 3:** Clinical Function Outcome Summary - KSS and KSFS.

**Author(s)**	**Outcomes Measured**		**Knee Society Score (SD)**						**Knee Society Function Score (SD)**					
			**Preop**	**Range**	**Postop/Final**	**Range**	**Improvement**	**Range**	**Preop**	**Range**	**Postop/Final**	**Range**	**Improvement**	**Range**
Anderson et al. (20)	KSS, ROM, survivorship		33 (−)	−	90 (−)	−	57 (−)	−	**Not performed**					
Bae et al. (21)	KSS, WOMAC, Kujala score, Feller scoring system, ROM, radiology, survivorship	**MP Group**	59.9 (7.5)	−	90.0 (6.6)	−	30.5 (11.4)	−	53.3 (7.1)	−	85.6 (8.5)	−	32.3 (10.8)	−
		**PS Group**	59.6 (8.3)	−	89.0 (6.1)	−	29.4 (10.7)	−	57.1 (8.6)	−	87.0 (6.9)	−	29.9 (11.8)	−
Brinkman et al. (22)	KSS, WOMAC, ROM, radiology, survivorship		33.5 (−)	12–91	84.0 (−)	33−100	50.5 (−)	9−21	50 (−)	15−90	80.0 (−)	45−100	30.0 (−)	10−30
Chinzei et al. (23)	KSS, radiology		36.2 (−)	0–65	92.1 (−)	65–100	55.9 (−)	35–65	31.4 (−)	0−75	73.4 (−)	45−100	22.0 (−)	25−75
Cho et al. (24)	KSS, ROM, radiology		61.5 (7.9)	−	90.4 (8.8)	−	28.9 (−)	−	57.8 (8.3)	−	84.8 (7.4)	−	27.0 (−)	−
Fan et al. (25)	ROM, KSS, radiology, survivorship		30.5 (2.3)	0–73	91.1 (1.3)	35–100	60.6 (−)	27–35	36.7 (1.7)	15−70	82.3 (1.7)	20−100	45.6 (−)	5−30
Hossain et al. (26)	KSS, WOMAC, OKS, SF-36, TKFQ, radiology	**MRK Group**	43.0 (13.6)	14–67	76.3 (15.5)	52–100	−	−	44.6 (15.3)	5−70	71.4 (15.8)	50−100	−	−
		**PFC Group**	48.4 (15.9)	11–88	68.6 (20.4)	40–99	−	−	47.9 (20.3)	5−95	68.0 (24.8)	10−100	−	−
Iida et al. (27)	ROM, KSS, radiology, survivorship		14 (13)	−	90 (10)	−	−	−	47 (13)	−	76 (22)	−	−	−
Ishida et al. (28)	KSS, ROM, KSFS, UCLA	**MP Group**	34.0 (−)	6–68	89 (−)	63–96	Median = 49 (−)	−	40 (−)	5−70	65 (−)	10–100	Median = 20 (−)	−
		**DH Group**	36.0 (−)	21–68	85 (−)	53–99	Median = 55 (−)	−	45 (−)	5−70	65 (−)	10–95	Median = 25 (−)	−
Karachalios et al. (29)	ROM, KSS, WOMAC, SF - 12, OKS, survivorship		31.6 (−)	10–70	91.3 (−)	70–100	−	−	42.9 (−)	5−60	80.9 (−)	35–100	−	−
Kim et al. (30)	ROM, KSS, HSSKS, radiology, survivorship	**MP Group**	29 (−)	2–50	87 (−)	70–100	−	−	45 (−)	20−60	80 (−)	30–100	−	−
		**PFC Group**	28 (−)	0–50	94 (−)	84–100	−	−	45 (−)	20−60	86 (−)	30–100	−	−
Moonot et al. (31)	OKS, KSS, IKS, radiology, ROM under fluoroscopy		−	−	95 (3)	86–98	−	−	−	−	99 (2)	94–100	−	−
Pritchett (32)	KSS, ROM, “which knee feels better”, radiology	**ACL Group**	38.6 (−)	−	92.6 (−)	−	54.0 (−)	−	41.9 (−)	−	76.7 (−)	−	34.8 (−)	−
		**MP Group**	40.3 (−)	−	93.2 (−)	−	41.9 (−)	−	46.1 (−)	−	75.2 (−)	−	29.1 (−)	−
		**PCL Group**	47.9 (−)	−	89.8 (−)	−	45.3 (−)	−	44.7 (−)	−	71.3 (−)	−	26.6 (−)	−
		**PS Group**	45.8 (−)	−	91.7 (−)	−	45.9 (−)	−	47.8 (−)	−	74.1 (−)	−	26.3 (−)	−
Pritchett (17)	KSS, ROM, “which knee feels better”	**ACL - PCL Group**	−	−	92.6 (−)	−	−	−	−	−	76.7 (−)	−	−	−
		**PCL Group**	−	−	90.8 (−)	−	−	−	−	−	71.3 (−)	−	−	−
		**PS Group**	−	−	91.7 (−)	−	−	−	−	−	74.1 (−)	−	−	−
		**MB Group**	−	−	92.4 (−)	−	−	−	−	−	81.1 (−)	−	−	−
		**MP Group**	−	−	94.2 (−)	−	−	−	−	−	80.4 (−)	−	−	−
Schmidt et al. (33)	KSS, ROM, survivorship		67.1 (−)	−	95.5 (−)	−	28.4 (−)	−	**Not performed**					
Vecchini et al. (36)	KSS, ROM, radiology, survivorship		28.3 (1.12)	−	73.2 (0.92)	−	−	−	49.1 (1.24)	−	78.9 (1.44)	−	−	−
Youm et al. (37)	ROM, KSS, WOMAC, radiology		46.6 (−)	34–66	87.4 (−)	73–97	40.8 (−)	−	38.6 (−)	25–45	82.0 (−)	63–100	43.4 (−)	−

SD, Standard Deviation; M, Male; F, Female; BMI, Body Mass Index; kg.m2, kilogram per meter squared; DH, Double High; MB, Mobile Bearing; MP, Medial Pivot; PS, Posterior Stabilized.

**Table 4:** Summary of Clinical Outcome Using Radiographic Parameters.

**Author(s)**		**Radiology Parameters**											
		**Preop Coronal Alignment; ° (SD)**	**Range; °**	**Postop Coronal Alignment; ° (SD)**	**Range; °**	**Tibial Component Slope; ° (SD)**	**Range;º**	****	**Range; °**		Range; °		**Range; °**
Bae et al. (21)	**MP Group**	varus 4.1 (4.3)	−	valgus 5.6 (3.0)	−	−	−	7.0 (3.4)	−	3.2 (2.4)	−	−3.8 (3.7)	−
	**PS Group**	varus 5.0 (5.6)	−	valgus 5.5 (2.7)	−	−	−	5.4 (4.8)	−	1.9 (3.0)	−	−3.6 (5.4)	−
Chinzei et al. (23)		varus 10.7 (−)	−9–31	valgus 1.4 (−)		−	−	−	−	−	−	−	−
Cho et al. (24)		varus 9.2 (6.5)	−	valgus 5.3 (2.7)	−	−	−	−	−	−	−	−	−
Fan et al. (25)		−	−	−	4–8	−	−	−	−	−	−	−	−
Hossain et al. (26)	**MRK Group**	−	−	−	−	−	−	−	−	−	−	−	−
	**PFC Group**	−	−	−	−	−	−	−	−	−	−	−	−
Iida et al. (27)		varus 10.0 (7.3)	−	valgus 6.0 (3.0)	−	−	−	−	−	−	−	−	−
Ishida et al. (28)	**MP Group**	varus 12 (−)	1–21	varus 1 (−)	−2–5	−	−	−	−	−	−	−	−
	**DH Group**	varus 11 (−)	1–20	varus 1 (−)	−1–5	−	−	−	−	−	−	−	−
Kim et al. (30)	**MP Group**	varus 5 (−)	1–14	valgus 5.0 (−)	0–8	−	−	−	−	4 (-)	−13–26	−	−
	**PFC Group**	varus 6 (−)	2–16	valgus 6.0 (−)	0–7	−	−	−	−	3 (-)	−18–20	−	−
Moonot et al. (31)		−	−	valgus 7.0 (2)	4–11	2 (3)	−4–4	−	−	−	−	−	−
Pritchett (32)		−	−	−	1–7	−	−	−	−	−	−	−	−
Pritchett (17)		−	−	−	1–7	−	−	−	−	−	−	−	−
Vecchini et al. (36)		−	−	−	−	−	−	−	−	−	−	−	−
Youm et al. (37)		−	varus 4.6 (4.5)	valgus 5.8 (2.4)	−	−	−	−	−	−	−	−	−

Author(s)		Radiology Parameters							
		Mean α angle; º (SD)	Range;º	Mean β angle; º (SD)	Range;º	Mean γ angle;º (SD)	Range;º	Mean δ angle; º (SD)	Range;º
Bae et al. (21)	**MP Group**	95.3 (2.6)	−	90.1 (2.1)	−	3.0 (2.1)	−	84.8 (2.9)	−
	**PS Group**	94.8 (3.6)	−	90.2 (2.0)	−	3.1 (2.1)	−	88.2 (3.4)	−
Chinzei et al. (23)		−	−	−	−	−	−	−	−
Cho et al. (24)		95.6 (3.7)	−	89.7 (2.2)	−	2.5 (3.6)	−	81.6 (2.8)	−
Fan et al. (25)		−	−	−	−	−	−	−	−
Hossain et al. (26)	**MRK Group**	95.6 (3.9)	80–100	88.4 (1.9)	84–92	2.4 (2.7)	−5–6	88.7(4.3)	78–99
	**PFC Group**	95.5 (3.5)	84–101	89.2 (2.5)	83–93	3.3 (4.7)	−5–15	87.4(2.8)	80–93
Iida et al. (27)		95.3 (2.9)	−	90.1 (2.0)	−	2.2 (2.8)	−	84.6 (11.7)	
Ishida et al. (28)	**MP Group**	−	−	−	−	−	−	−	
	**DH Group**	−	−	−	−	−	−	−	
Kim et al. (29)	**MP Group**	96 (−)	91–101	89 (−)	80–98	3 (−)	−2–8	84 (−)	77–95
	**PFC Group**	97 (−)	90–101	89 (−)	83–95	2 (−)	−3–6	85 (−)	77–91
Moonot et al. (30)		Not reported	−	Not reported	−	Not reported	−	Not reported	−
Pritchett (32)		−	−	−	−	−	−	−	−
Pritchett (17)		−	−	−	−	−	−	−	−
Vecchini et al. (36)		Not reported	−	94 (−)	87–96	Not reported	−	Not reported	−
Youm et al. (37)		96.2 (2.1)	−	89.1 (1.7)	−	2.5 (1.5)	−	84.4 (2.7)	−

DH, Double High; MP, Medial Pivot; PS, Posterior Stabilized.

**Table 5:** Clinical Function Outcome Summary - WOMAC and OKS.

**Author(s)**		**WOMAC Score (SD)**						**OKS (SD)**					
		**Preop**	**Range**	**Postop/Final**	**Range**	**Improvement**	**Range**	**Preop**	**Range**	**Postop/Final**	**Range**	**Improvement**	**Range**
Bae et al. (21)	**MP Group**	32.9 (4.8)	−	14.3 (5.7)	−	18.5 (6.6)	−	Not performed					
	**PS Group**	35.1 (4.1)	−	15.8 (5.7)	−	19.3 (6.4)	−						
Brinkman et al. (22)		34 (−)	12–86	22 (−)	1–76	−	−	Not performed					
Hossain et al. (26)	**MRK Group**	56.0 (17.3)	14–93	27.1 (13.4)	8–50	−	−	41.6 (7.5)	25–56	26.2 (9.1)	16–44	−	−
	**PFC Group**	53.8 (19.4)	15–88	32.9 (23.1)	13–41	−	−	41.7 (8.9)	23–56	29.1 (7.0)	13–41	−	−
Karachalios et al. (29)		30.8 (−)	15–54	79.2 (−)	43–95	−	−	44.4 (−)	36–48	22.6 (−)	15–40	−	−
Moonot et al. (31)		Not performed						−	−	17 (3)	12–13	−	−
Shimmin et al. (35)		Not performed						−	−	39 (−)	11–48	−	−
Youm et al. (37)		54.8 (−)	−	18.3 (−)	−	−	−	Not performed					

MP, Medial Pivot; PS, Posterior Stabilized; OKS, Oxford Knee Score; WOMAC, Western Ontario and McMaster Universities Arthritis Index.

**Table 6:** Clinical Function Outcome Summary - SF- 36 and TKFQ.

**Author(s)**		**SF −36 (SD)**						**TKFQ (SD)**					
		**Preop**	**Range**	**Postop/Final**	**Range**	**Improvement**	**Range**	**Preop**	**Range**	**Postop/Final**	**Range**	**Improvement**	**Range**
Hossain et al. (26)	**MRK Group**	Physical = 26.0 (6.8); mental = 49.9 (12.5)	Physical: 8.5–41.4; mental: 27.5–70.4	Physical = 39.5 (12.8); mental = 46.3 (8.3)	Physical: 15.6–61.4; mental: 28.5–63.1	−	−	2.6 (1.5)	0–5.6	5.9 (1.0)	3.9–7.4	−	−
	**PFC Group**	Physical = 26.7 (7.0); mental = 51.3 (10.4)	Physical: 17–46.9; mental: 30.7–70.8	Physical = 32.8 (12.6); mental = 43.4 (14.1)	Physical: 10.9–55.6; mental: 18.4–65.2	−	−	3.1 (1.6)	0.6–5.4	5.1 (1.5)	2.4–6.9	−	−
Karachalios et al. (29)		SF 12 (physical) = 26.6 (−)	19–40.5	SF 12 (physical) = 47 (−)	35–56.6	−	−	−					

SF - 36, Short Form 36; TKFQ, Total Knee Function Questionnaire.

**Table 7:** Clinical Function Outcome Summary - VR-12, KOOS, Kujuala, Fellers and EuroQoL.

**Author(s)**				**VR-12 Score (SD)**				**KOOS Score (P/S/FuADL/FuSR/QoL) (SD)**						**Feller Score (SD)**					
		Preop	**Range**	**Postop/Final**	**Range**	**Improvement**	**Range**	**Preop**	**Range**	**Postop/Final**	**Range**	**Improvement**	**Range**	**Preop**	**Range**	**Postop/Final**	**Range**	**Improvement**	**Range**
**Shimmin et al.(35)**		−	−	Mental scale = 50 (−); physical scale = 45 (−)	Mental: 14–67; physical: 28–56	−	−	−	**−**	Pain = 92 (−)	**−**	**−**	**−**	**Not performed**					
										Symptoms = 91 (−)	**−**	**−**	**−**						
										Function in Daily Living Activities = 91 (−)	**−**	**−**	**−**						
										Function in Sports and Recreation = 62 (−)	**−**	**−**	**−**						
										Quality of Life = 78 (−)	**−**	**−**	**−**						
**Bae et al.(21)**	**MP Group**	**Not performed**						**Not performed**						18.9 (2.6)	−	26.6 (2.4)	−	7.7 (3.1)	−
	**PS Group**													18.6 (2.5)	−	26.6 (1.9)	−	7.6 (2.7)	−

**Author(s)**		**Kujala Scores (SD)**						**EuroQol (SD)**					
		**Preop**	**Range**	**Postop/Final**	**Range**	**Improvement**	**Range**	**Preop**	**Range**	**Postop/Final**	**Range**	**Improvement**	**Range**
**Shimmin* *et al. (35)**		**−**	**−**	79 (−)	58–100	**−**	**−**	**−**	**−**	1.21 (–)	1–2	**−**	**−**
									
									
**Bae* *et al. (21)**	**MP Group**	50.4 (6.7)	−	80.1 (7.5)	**–**	29.8 (9.9)	–	Not performed					
	**PS Group**	49.3 (5.4)	−	77.9 (6.4)	**–**	28.6 (7.2)	–						

KOOS, Knee Injury and Osteoarthritis Outcome Score; VR-12, Veterans Rand - 12. MP, Medial Pivot; PS, Posterior Stabilized; EuroQol, European Quality of Life Scale.

**Table 8:** Clinical Function Outcome Summary – UCLA.

**Author(s)**	**UCLA Score (SD)**						
		**Preop**	**Range**	**Postop/Final**	**Range**	**Improvement**	**Range**
Ishida et al. (28)	** MP Group**	3 (−)	2–8	1 (−)	2–8	−	−
	**DH Group**	3 (−)	1–8	1 (−)	1–8	−	−

MP, Medial Pivot; DH, Double High; UCLA, University of California, Los Angeles.

### Knee Society Score (KSS) and Knee Society Functional Score (KSFS)

Sixteen studies reported mean postoperative KSS value to be in the “excellent” range (80 ~ 100 points) (17, 20–25, 27–33, 37). Two studies reported the postoperative KSS values to be in the range that is “good” (70 to 79 points) (26, 36). In their study, Hossain et al. described the postoperative KSS value to be “good” (70 ~ 79 points) in the medial stabilized group, however did not grade the KSS value (69.4 points) in the posterior stabilized (PS) group (26).

**Figure 4:**
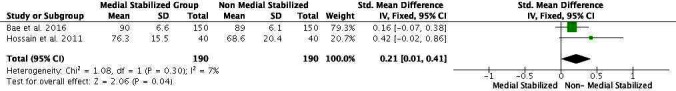
Forest plot of final KSS value of medial stabilized group and non-medial stabilized group (SD: Standard Deviation; CI: Confidence Interval).

**Figure 5:**
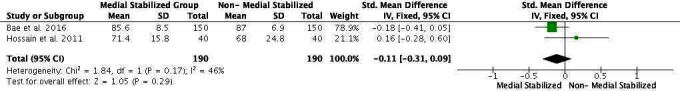
Forest plot of final KSFS value of medial stabilized group and non-medial stabilized group (SD: Standard Deviation; CI: Confidence Interval).

The final mean KSS value in the medial stabilized group was 89.92 points, compared to 90.76 points in the non-medial stabilized group. As shown in Figure 4, the analysis showed the standard mean difference (SMD) between the two groups to be statistically significant: (SMD 0.21; 95% CI: 0.01 to 0.41; *p* = 0.04). The final mean KSFS value in medial stabilized group was 79.68 points and 76.18 points in the non-medial stabilized group, the mean difference between the two groups was statistically insignificant as shown in Figure 5 (SMD: −0.11; 95% CI: −0.31 to 0.09; *p* = 0.29).

### Western Ontario and McMaster Universities Arthritis Index (WOMAC)

**Figure 6:**
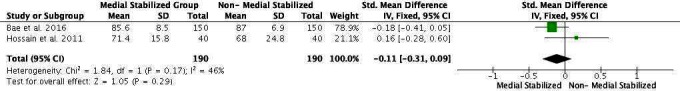
Forest plot of final WOMAC value of medial stabilized group and non-medial stabilized group (SD: Standard Deviation; CI: Confidence Interval).

Five studies used WOMAC as one of the modalities to quantify clinical function (21, 23, 27, 30, 38). The final mean WOMAC values were 23.73 and 19.40 for the medial stabilized and non-medial stabilized groups respectively, with the mean difference between the two groups being statistically significant and favoring the medial stabilized group as shown in Figure 6 (SMD: −0.27; 95% CI: −0.47 to −0.07; *p* = 0.009).

**Figure 7:**
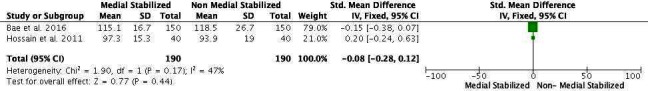
Forest plot of preoperative Knee ROM of medial stabilized group and non- medial stabilized group (SD: Standard Deviation; CI: Confidence Interval).

**Figure 8:**
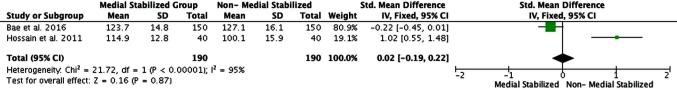
Forest plot of Knee ROM at final follow-up of medial stabilized group and non-medial stabilized group (SD: Standard Deviation; CI: Confidence Interval).

The preoperative mean knee ROM value in the medial stabilized group across all studies was 107.89°, and 112.76° in the non-medial stabilized group. The mean difference between the groups was statistically insignificant (SMD: −0.08; 95% CI: −0.28 to 0.12, *p* = 0.44, Figure 7). The final mean knee ROM value in the medial stabilized group across all studies was 116.29°, compared to 117.90° in the non-medial stabilized group, the mean difference between the two groups was statistically insignificant (SMD: 0.02; 95% CI: −0.19 to 0.02; *p* = 0.87, Figure 8).

The *I*^2^ values (heterogeneity) varied greatly in the parameters examined: from 0% for final mean WOMAC value, 4% for final mean KSS value, 29% for final mean KSFS value, 46% for preoperative mean knee ROM, and observed to be 95% for the final mean knee ROM value.

## Discussion

### Summaryose

The aim of this review was to determine whether differences exist in clinical outcome measures between patients with a medial stabilized TKJR construct and those with non-medial stabilized designs. We found that there is statistically significant mean difference in the mean final WOMAC values favouring the medial stabilized group, and statistically significant difference in the final mean KSS values favouring the non-medial stabilized group. The results may be explained that the clinician derived component of KSS, and a more specific set of questions from WOMAC assessing levels of functional limitations may have influenced the results in contrasting fashion in the analysis. The KSS is unique in that it contains both patient reported, and surgeon reported components to express and define the clinical and subjective status of the knee. It recognises and takes objective findings that are known to influence the functional outcomes (38). It has the advantage of quantifying objective clinical parameters such as range of motion of the knee, fixed flexion deformity, alignment and ligamentous laxity and integrate into the outcome measure itself. The subjective component is self- administered by the patient, and focuses on the symptoms, level of patient satisfaction, and patient expectations (39). WOMAC is a patient reported outcome measure that inspects the characteristics of pain, stiffness and function. It has five items for pain (score range 0–20), two for stiffness (score range 0–8), and 17 for functional limitation (score range 0–68) (40). The pain component questions explore the level of pain at rest, standing, pain walking, and pain climbing or descending stairs, and the physical functioning component employing 17 questions examining the level of limitations specifically through everyday activities from ascending and descending stairs, standing, rising from lying, sitting, bending to floor, getting in and out of a car, putting on socks, toileting and baths. The KSS does account for the issue of pain not only with the two visual analogue scales from 1 to 10 for level of pain when walking on flat ground, and climbing and descending stairs, but also it asks the patients their level of satisfaction with regards to pain at rest, and assesses the level of functional limitations through questions covering tasks of getting out of bed, perform household duties and leisurely duties. The questions however are not as comprehensive when compared to those in WOMAC.

It is important to be able to relate the statistical significance uncovered in the context of the clinical setting. For example, the mean difference in the mean WOMAC values between the two groups was statistically significant (4.33 points), one would not be able to draw a clinically meaningful interpretation of this statistically significant numerical difference. In their prospective study, Escobar et al. concluded that the minimal change required in WOMAC scores to show a clinically significant difference was 15 points (41). In our analysis, the mean difference in the final mean KSS was statistically significant, however the difference of 0.84 points between the groups would not be significant in the clinical setting.In a recent retrospective study, the authors collected KSS, KSFS as well as OKS in 550 patients prior to their respective TKJR operations, and two years after the operations. They identified the Minimal Clinically Important Difference (MCID) for KSS to be between 5.3 to 5.9 points, and MCID for KSFS to be between 6.1 to 6.4 points (39). In our analysis, the mean differences of preoperative, and final knee ROM between the two groups were 4.87 degrees and 1.61 degrees respectively, they were observed to be statistically insignificant. These mean difference values would also be insignificant in the clinical setting.

In this analysis, the *I*^2^ values ranged from 0% for the final mean difference WOMAC values to 95% for the final mean difference knee ROM values. *I*^2^ describes the proportion of variability in percentage scale that is due to between-studies variance rather than within-study sampling error, and it assesses the level of consistency of results produced across studies in a meta-analysis (42). The* I^2^* value of zero seemed implausible, unless it is known that the studies were performed in the exact same way, and involve individuals sampled from the same population (43). High *I*^2^ values could be caused by factors such as small sample sizes of the individual studies in the analysis, the non-randomized design of the studies analyzed, or unmeasured variables such as differences in population sampled or implementation of the respective study protocols (42). The variable *I^2^* values in this analysis would suggest that there was a different magnitude of unexplained between-studies variance, thus making the final pooled estimate results not representative of the studies analyzed, and one cannot determine the applicability of the findings produced in the analysis.

There are contrasting reports in the relative degrees of efficacy of medial stabilized TKJR construct comparing to other types of construct designs in the literature. An insight into Kim et al. (31) who enrolled participants requiring bilateral TKJRs, implanted a medial stabilized TKJR system (ADVANCE^®^) and a Depuy PFC^®^ mobile bearing prosthesis in the other, reported the KSS values were in the “excellent” range for the medial stabilized TKJR knees, but noted that these values were statistically significantly lower than those for the PFC^®^ system in the contralateral knee (mean final KSS value of 87 points and 94points respectively, *p* = 0.02). The authors also reported that the postoperative mean knee ROM measurements were consistently better in the PFC^®^ knees than the medial stabilized knees at three months (126° to 98°, *p* < 0.05), one year (128° to 110°, *p* < 0.05), and at final follow up 2.6 years after the operations (127° to 115°, *p* < 0.05). Similarly, Shakespeare et al. (35), who compared 261 knees replaced with the medial stabilized TKJR system to 288 replaced with a posterior stabilized TKJR system, suggested that while there was no significant difference in the mean knee flexion angle between the two implants, the regression analysis of individual knees revealed a small but statistically significant greater loss of knee flexion (2.9°, β coefficient 2.923, *p* = 0.007) in the medial stabilized group 12 months postoperatively.

By using a matched pair analysis, Bae et al. (21) compared the clinical and radiographic results between the participants implanted with medial stabilized and posterior stabilized prostheses. They noted that the extent of improvement in clinical scores, radiographic results, and patellofemoral symptoms were statistically similar between their medial stabilized and posterior stabilized cohorts. In their study, Hossain et al. (27) conducted a single centre, single blinded randomised controlled trial enrolling 82 participants to compare the medial stabilized knee construct prosthesis (MRK^™^) to a conventional fixed bearing PS TKJR design construct. The investigators noted a statistically significant difference in the final mean knee ROM between the two groups favouring the medial stabilized design at one year (98.2° for PS group and 115.5° for MRK^™^, *p* < 0.0001) and two years postoperatively (100.1° for PS, and 114.9° for MRK^™^, *p* < 0.0001) (27). Furthermore, the authors found the medial stabilized group had better physical component scores of Short Form 36 (SF-36) (32.6 points for PS group, and 40.3 points for MRK^™^ group at one year, *p* = 0.008; 32.8 points for PS group and 39.5 points for MRK^™^ group after two years, *p* = 0.02), and better Total Knee Functional Questionnaire (TKFQ) values at one year, and two years after the knee operations (27).

### Limitations

The study is not without limitations. Only data from English language peer reviewed journals were included in this systematic review. This limitation can potentially omit some relevant data presented in non-English language journals, as well as data from unpublished trials. The absence of the critical statistical detailsuch as SD in most studies included seriously handicapped the extent of the meta- analysis, compromising quality of the results. The ability to make any firm conclusions, given the small size of sample data, the lack of comparative randomised data, and the varying degrees of heterogeneity between studies, is limited. The standard mean differences of each parameters examined may not truly reflect how the medial stabilized TKJR design truly measures up against other prosthetic designs in terms of clinical performance. The present review specifically aimed not to examine the survivorship of the medial stabilized construct design implants in the studies, as it has been previously explored in recent meta analyses and literature reviews (1, 44–46).

## Conclusion

Within the confines of the available studies, we found statistically significant mean differences in the mean final WOMAC values favouring the medial stabilized group, and statistically significant differences in the final mean KSS values favouring the non-medial stabilised group. There were no statistically significant differences in the final mean KSFS values, preoperative mean knee ROM values and final mean knee ROM values between the two groups. Based on the analysis of the literature review, a firm conclusion cannot be reached regarding the comparative clinical performance of the medial stabilized TKJR construct.

There are different reports of clinical performance of the medial stabilized construct design when compared to other TKJR designs, and there is a lack of data evaluating clinical function and fluoroscopic analysis of the medial stabilized construct design compared to other construct designs in the context of a randomised controlled trial. The significant heterogeneity in the outcomes examined in this review suggests that further research is needed to quantify differences in knee biomechanics and clinical outcome measures between the most commonly used TKJR designs.

## Author Contributions

TY performed the literature search and meta-analysis in its entirety, as well as writing this article. PC is the senior supervisor in the PhD project, MD and MP provided guidance and expertise in the clinical and biomechanical aspects of the PhD project respectively. All authors have read and approved the final submitted manuscript.

## Conflict of Interest Statement

TY received a Scholarship Stipend funded by Medacta International SA in 2015. The sponsor has no involvement in the day to day running of the project nor data analysis. The other authors declare that the research was conducted in the absence of any commercial or financial relationships that could be construed as a potential conflict of interest.
